# OSucs: An Online Prognostic Biomarker Analysis Tool for Uterine Carcinosarcoma

**DOI:** 10.3390/genes11091040

**Published:** 2020-09-03

**Authors:** Yang An, Qiang Wang, Fengjie Sun, Guosen Zhang, Fengling Wang, Lu Zhang, Yanan Li, Weinan Ren, Wan Zhu, Yongqiang Li, Shaoping Ji, Xiangqian Guo

**Affiliations:** 1 Department of Predictive Medicine, Institute of Biomedical Informatics, Cell Signal Transduction Laboratory, Bioinformatics Center, Henan Provincial Engineering Center for Tumor Molecular Medicine, Kaifeng Key Laboratory of Cell Signal Transduction, School of Basic Medical Sciences, School of Software, Henan University, Kaifeng 475004, China; anyang@henu.edu.cn (Y.A.); qiangwang@henu.edu.cn (Q.W.); 13167945419@163.com (F.S.); zhangguosen1989@126.com (G.Z.); wangfl@henu.edu.cn (F.W.); zhanglu9128@126.com (L.Z.); 1710191066@vip.henu.edu.cn (Y.L.); R1220255343@163.com (W.R.); liyongqiang@vip.henu.edu.cn (Y.L.); shaopingji@henu.edu.cn (S.J.); 2 Department of Anesthesia, Stanford University, Stanford, CA 94305, USA; ms.wanzhu@gmail.com

**Keywords:** uterine carcinosarcoma, prognostic biomarker, gene expression profiling, survival analysis tool, molecular subtype

## Abstract

Background: Uterine carcinosarcoma (UCS) is a type of rare and aggressive tumor. The standard treatment for UCS involves surgical treatment followed by radiochemotherapy. Clinical outcomes of UCS patients are poor due to high metastasis and relapse rate. Therefore, new targeted therapy strategies for UCS are needed. Because UCS is highly heterogenous, it is critical to identify and develop prognostic biomarkers to distinguish molecular subtypes of UCS for better treatment guidance. Methods: Using gene expression profiles and clinical follow-up data, we developed an online consensus survival analysis tool named OSucs. This web tool allows researchers to conveniently analyze the prognostic abilities of candidate genes in UCS. Results: To test the reliability of this server, we analyzed five previously reported prognostic biomarkers, all of which showed significant prognostic impacts. In addition, ETV4 (ETS variant transcription factor 4), ANGPTL4 (Angiopoietin-like protein 4), HIST1H1C (Histone cluster 1 H1 family member c) and CTSV (Cathepsin V) showed prognostic potential in a molecular subtype-specific manner. Conclusion: We built a platform for researchers to analyze if genes have prognostic potentials in UCS.

## 1. Introduction

Uterine carcinosarcoma (UCS), also known as a malignant mixed Müllerian tumor (MMMT), is a type of rare, highly aggressive, biphasic malignant tumor with carcinomatous and sarcomatous components [1]. Although UCS has a relatively low annual incidence rate of 5.1–6.9 per 1,000,000 women and accounts for less than 5% of uterine cancer (UC), it has contributed up to 30% of uterine cancer mortality due to its highly aggressive nature [2]. Treatment of UCS mainly relies on surgery, i.e., lymphadenectomy. Although surgical resection and subsequent radiochemotherapy improve the overall survival rate of patients, the five-year survival rate is still low (18–39%) [3]. In recent years, a few prognostic biomarkers based on serum or protein level detection have been reported, including CA125, CA15-3, CEA, and CA19-9 [4,5,6], but due to the molecular heterogeneity of tumors, more biomarkers at mRNA level are required. To facilitate the discovery of prognostic biomarkers, researchers need a platform to quickly evaluate potential prognostic biomarkers in multiple independent cohorts. In this study, we established an online web server named OSucs to examine the association between gene expression and survival for UCS patients. Specifically, users can expediently evaluate the prognostic value of the candidate gene of interest. The advantage of the OSucs server is that OSucs could perform the UCS molecular subtype-specific prognosis analysis [7].

## 2. Methods

### 2.1. Data Collection and Processing

Gene expression profiling data (RNA-seq, level-3, HiSeqV2) and clinical information of 57 cases of uterine carcinosarcoma were collected from The Cancer Genome Atlas (TCGA) database in 2016. Follow-up data were used to calculate survival values, including overall survival (OS), disease-specific survival (DSS), disease-free interval (DFI) and progression-free interval (PFI), based on a previous study [8], while one case (No. TCGA-QN-A5NN) was excluded because the survival values of this patient were 0. Thus, the sample size in OSucs was 56 cases.

### 2.2. Development of OSucs

**Figure 1:**
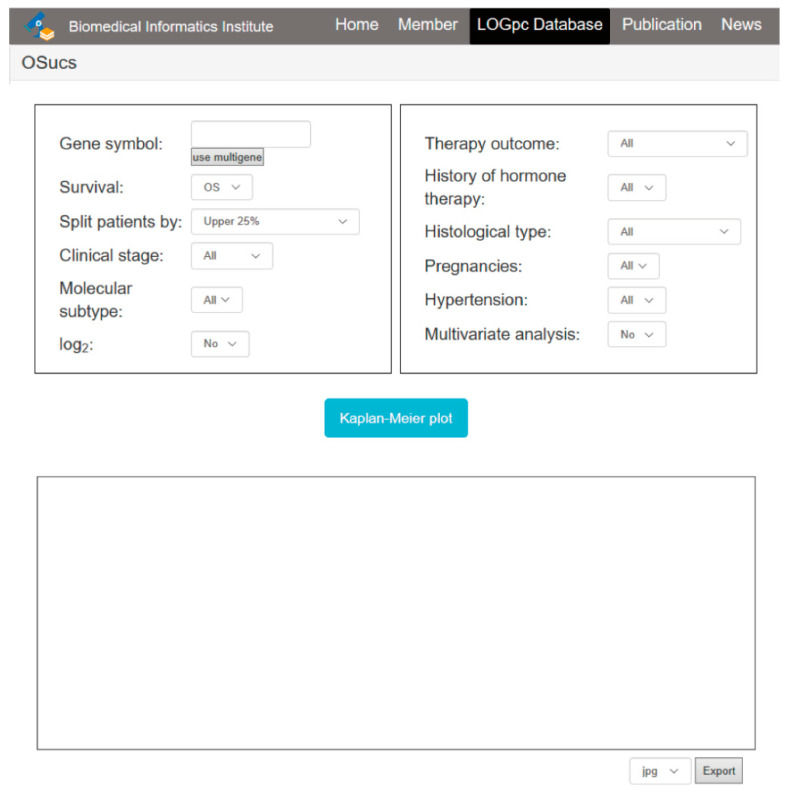
Screenshot of the main interface of OSucs at http://bioinfo.henu.edu.cn/UCS/UCSList.jsp.

The OSucs web server was hosted on a Tomcat (Apache, Minneapolis, MN, USA) server on a Windows system and operated by Java and R to handle the requests from users and return the analysis results to users. Gene expression profiling and clinical data were stored and managed by a SQL Server. The JDBC (Java Database Connectivity) package acted as connection middleware between Java and SQL Server. The Kaplan-Meier (KM) survival curves with hazard ratio (HR with 95% confidence interval) and *p* values were calculated by R packages ‘survival’ and ‘survminer’. The web server integrated with R application code seamlessly by using an ‘Rserver’ package. The web server uses the method coxph in the R ‘survival’ package to perform Cox regression analysis, and uses the ggsurvplot method in R ‘survminer’ and ‘ggplot2’ packages to construct the KM plot figure. Univariate and multivariate Cox regression analysis were applied to evaluate the prognostic values of the risk factors and the input gene. In addition, multigene analysis was implemented using gene expression level weighted with the regression coefficient, which was obtained from the univariate analysis. The formula is as follows: risk score = ∑ (Exp 1 × β1 + Exp 2 × β2 + … + Exp n × βn). OSucs can be accessed at http://bioinfo.henu.edu.cn/UCS/UCSList.jsp. The system architecture flow diagram is as described in previous reports [9,10,11,12,13,14,15,16,17], and a screenshot of the web server interface is presented in Figure 1.

### 2.3. Application of OSucs

First, users could input a gene symbol into the ‘Gene symbol’ box. If the gene symbol was not an official gene name, the ‘invalid input’ warning would be displayed. The ‘Split patients by’ dialog box provided eight options for users to categorize patients into two subgroups according to the expression level of the input gene. In addition, some clinical factors including ‘Molecular subtype’, ‘Histological type’, ‘Clinical stage’, ‘Therapy outcome’, ‘Pregnancies’, ‘Hormone therapy’ and ‘Hypertension’ were set as optional factors to further categorize patients. By clicking the ‘Kaplan-Meier plot’ button, the server would take the request and return the analysis result with graphically displayed HR, 95% CI and *p* value. However, when the sample size was less than four, the analysis could not be completed due to insufficient statistical power and the prompt “Number of UCS patients you analyzed in at least one of the groups is less than four in the dataset, thus no meaningful output returns” would be displayed.

### 2.4. Verification of Prognostic Biomarkers in OSucs

The prognostic abilities of previously reported biomarkers were verified by graphing the Kaplan-Meier plots in OSucs. Gene symbols of reported biomarkers were typed into the ‘Gene symbol’ input box individually, and the survival curve was obtained by clicking the ‘Kaplan-Meier plot’ button. The details, including ‘Gene symbol’, ‘cut-off’, ‘HR’, ‘*p* value’ and ‘prognostic outcome’, were listed to compare the prognostic abilities of these biomarkers between ‘In OSucs’ and ‘In reference’.

## 3. Results

### 3.1. Establishment and Application of OSucs

OSucs is a web platform for evaluating prognostic values of a candidate gene. The underlining pipeline was established by applying a Kaplan-Meier plot to present the association between gene of interest and survival rate. On this server, ‘Gene symbol’, ‘Survival’ and ‘Split patients by’ were set as the three main parameters (Figure 1). The general process was that an official gene name (such as NCBI authorized) would be expected in the gene symbol box. A red warning message would be given if the input was not an official gene symbol. Survival information could be analyzed by choosing the items of interest to users, including OS, DSS, DFI and PFI [8]. We analyzed patients’ statistics and showed that the median time of OS was 20.37 months, and the median time of DFI was 13.57 months (Table 1). Under ‘Split patients by’ option, the patients could be categorized by the expression level of the candidate gene (such as upper or lower 25%, 30% and 50%), and users could choose different thresholds (Figure 1). Further, various other options were available to group UCS patients of interest, including ‘Molecular subtype’, ‘Histological type’, ‘Clinical stage’, ‘Therapy outcome’, ‘Pregnancies’, ‘Hormone therapy’ and ‘Hypertension’ (Figure 1). Taking ‘Molecular subtype’ as an example, users could select molecular subtype (All, I or II) of UCS from a drop-down menu to evaluate the subtype-specific prognostic value of a candidate gene. By clicking the blue ‘Kaplan-Meier plot’ button, the association between the candidate gene and survival would be calculated by the OSucs server. As an output, the analysis results were graphically displayed as a survival curve and presented with *p* value and HR (with 95% confidence interval).

### 3.2. Survival Analysis of Clinicopathologic Characteristics of UCS Patients in OSucs

**Figure 2:**
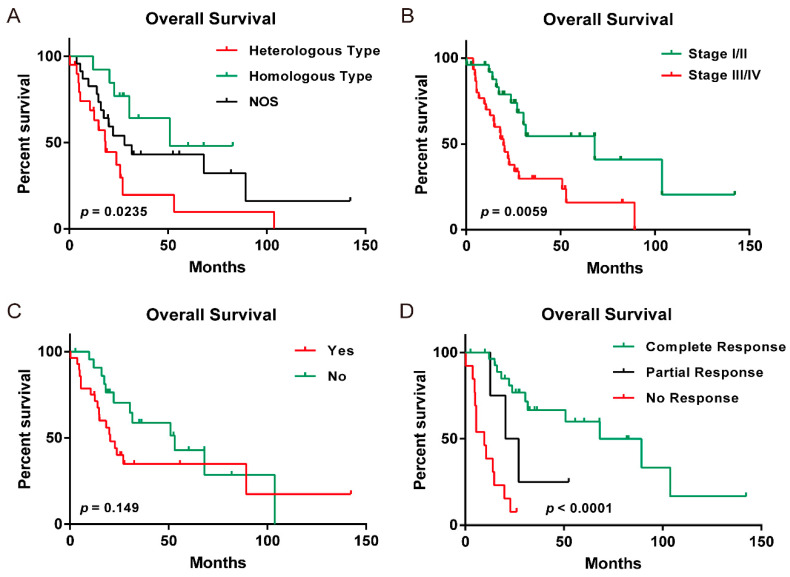
Survival analysis (overall survival, OS) of clinicopathologic characteristic of uterine carcinosarcoma (UCS) patients in OSucs. (**A**) Histological type, (**B**) clinical stage, (**C**) hypertension, (**D**) therapy outcome.

On the OSucs platform, UCS was stratified into two distinct molecular subtypes with different gene expression patterns and clinicopathologic characteristics according to a previous study [7]. Specifically, subtype I UCS was featured with cell adhesion and apoptosis pathways, while subtype II was characterized by myogenesis/muscle development pathways. The rationale of this molecular subtyping would be helpful for developing subtype-specific targeted therapy. In this study, we further analyzed the association between survival and clinicopathologic characteristics including molecular subtype. By analyzing the 56 UCS patients, we showed that molecular subtype I and subtype II patients accounted for 68% and 30% of all the UCS patients, respectively (Table 1). However, patients of different molecular subtypes had no survival differences (neither OS nor DFI; data not shown). The histological type of UCS was classified into heterologous type, homologous type or not otherwise specified (NOS) type, which accounted for 23%, 36% and 41% of the total patients, respectively (Table 1). By analyzing the clinical stages of the UCS patients, we found that stage I, II, III and IV patients accounted for 38%, 9%, 36% and 18% of the total patients, respectively (Table 1). It is worth mentioning that the histological type of UCS was significantly associated with OS, but not DSS, DFI and PFI, and the clinical stage of UCS was significantly associated with OS and DSS, but not DFI and PFI (Figure 2a,b and Figure S1a,b). Interestingly, the hypertension status of UCS patients was significantly associated with DSS and PFI, but not with OS and DFI (Figure 2c and Figure S1c). In addition, 50% of UCS patients were suffering from hypertension, while 41% were not (Table 1). By analyzing therapeutic outcome, UCS patients could be divided into complete, partial or no response to treatment, which accounted for 52%, 7% and 23% of total patients, respectively (Table 1). Notably, the therapy outcome of UCS patients was significantly associated with all the survival values (OS, DSS, DFI and PFI, *p* < 0.0001) (Figure 2d and Figure S1d). Nevertheless, neither history of hormone therapy nor pregnancies had a significant association with survival (data not shown).

### 3.3. Validation of Previously Reported UCS/UC Prognostic Biomarkers in OSucs

**Figure 3:**
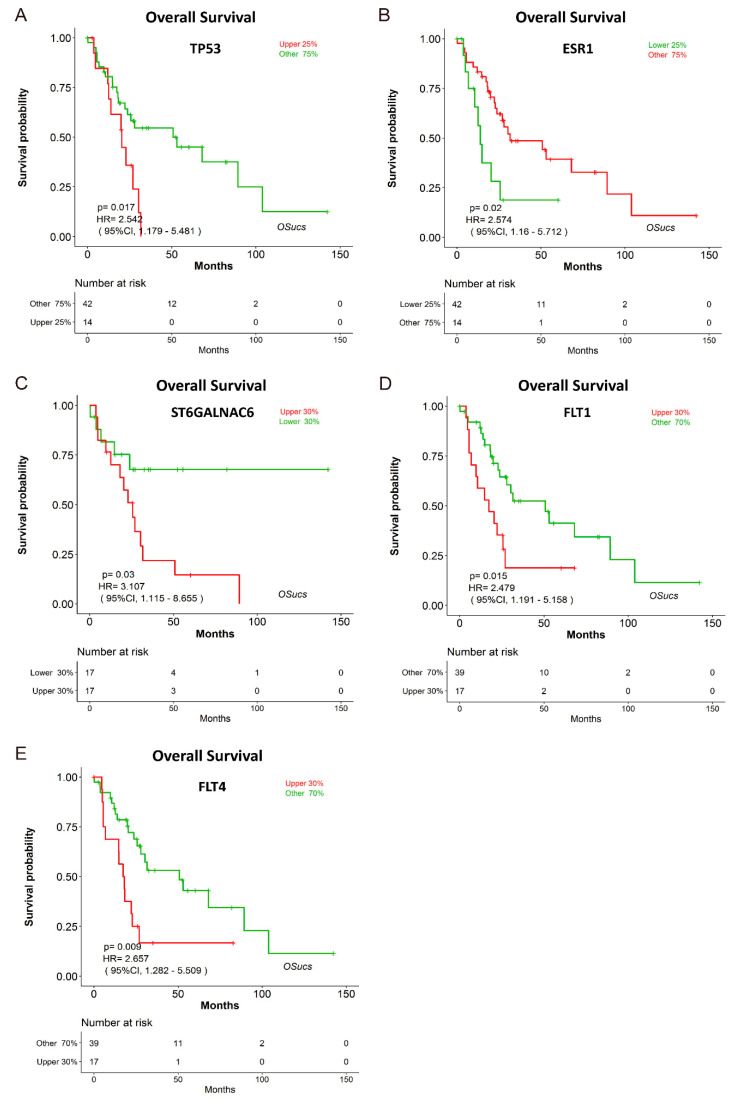
Validation of previous reported prognostic biomarkers in OSucs. Kaplan-Meier plots for (**A**) TP53, (**B**) ESR1, (**C**) ST6GALNAC6, (**D**) FLT1 and (**E**) FLT4 (OS).

To evaluate the prognostic analysis ability and reliability of the web server, we searched previous reported biomarkers for UCS prognosis in PubMed using the keywords of ‘uterine carcinosarcoma’ and ‘prognostic biomarker’. Thus, we evaluated the prognostic abilities of five reported prognostic biomarkers in OSucs, including p53 (encoded by TP53 gene [18]), ER (encoded by ESR1 gene), CA19-9 (encoded by ST6GALNAC6 gene), p-flt-1 (encoded by FLT1 gene) and VEGFR3 (encoded by FLT4 gene) (Table 2). As a result, all of these biomarkers have been verified in OSucs (Table 2, Figure 3, Figures S2 and S3). As previously reported [19,20,21,22], these genes were significantly associated with survival in OSucs, and the patients with elevated ESR1 expression have longer OS and DSS, while the patients with higher expression of TP53, ST6GALNAC6, FLT1 and FLT4 have shorter survival (Table 2, Figure 3, Figures S2 and S3).

### 3.4. Evaluation of Potential Prognostic Biomarkers for UCS Molecular Subtypes in OSucs

**Figure 4:**
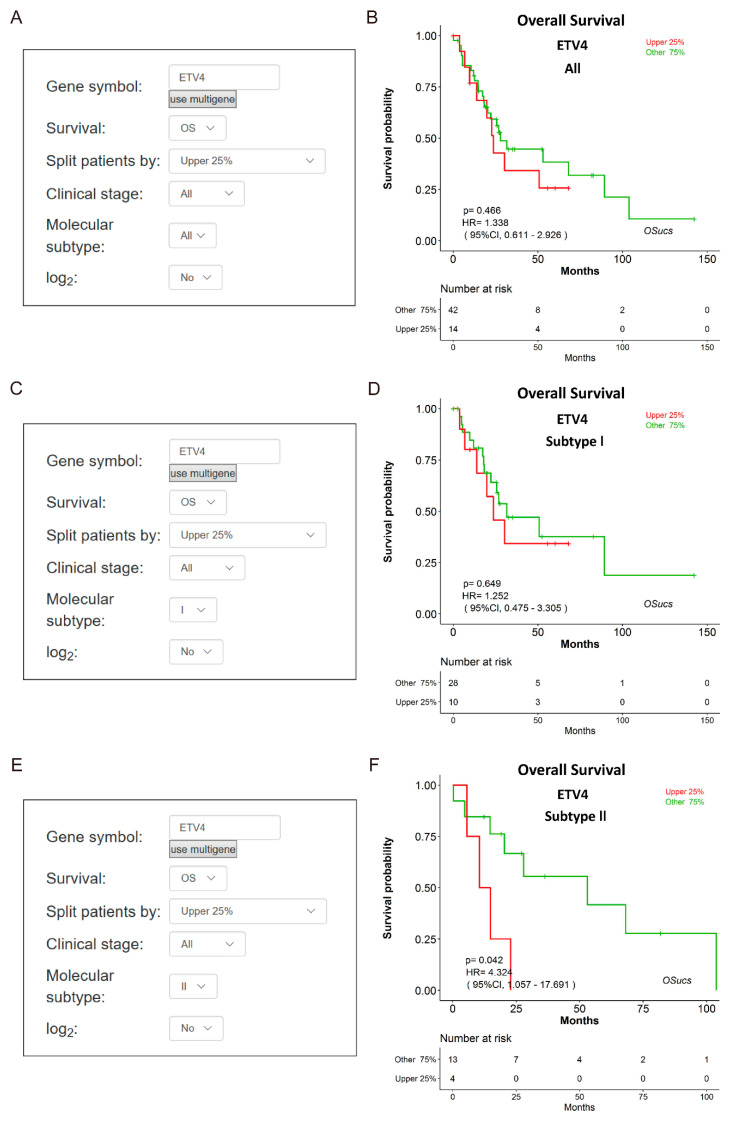
Evaluation of the prognostic value of ETV4 (ETS variant transcription factor 4) gene in OSucs. (**a**,**c**,**e**) Screenshots of molecular subtype selection in OSucs main interface. (**b**,**d**,**f**) Kaplan-Meier plots for ETV4 (OS) in All, Subtype I and Subtype II UCS, respectively.

**Figure 5:**
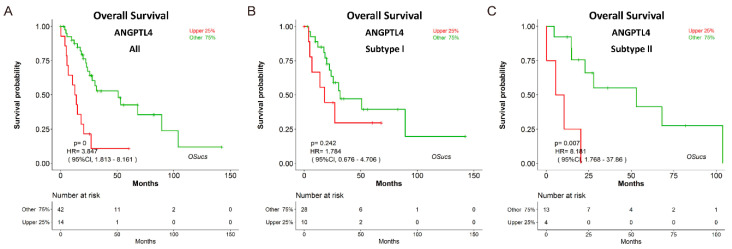
Evaluation of the prognostic value of angiopoietin-like protein (ANGPTL4) in OSucs. Kaplan-Meier plots for ANGPTL4 in (**A**) All, (**B**) Subtype I, and (**C**) Subtype II UCS, respectively. *p* = 0 denotes *p* < 0.001.

**Figure 6:**
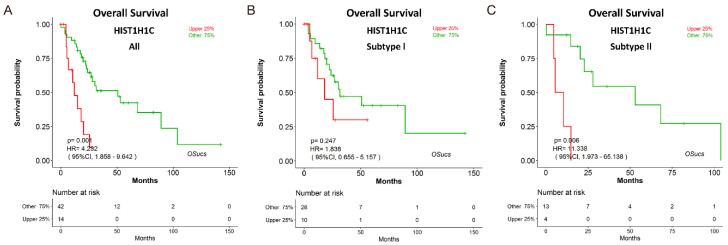
Evaluation of the prognostic value of histone cluster 1 H1 family member c (HIST1H1C) in OSucs. Kaplan-Meier plots for HIST1H1C in (**A**) All, (**B**) Subtype I, and (**C**) Subtype II UCS, respectively.

**Figure 7:**
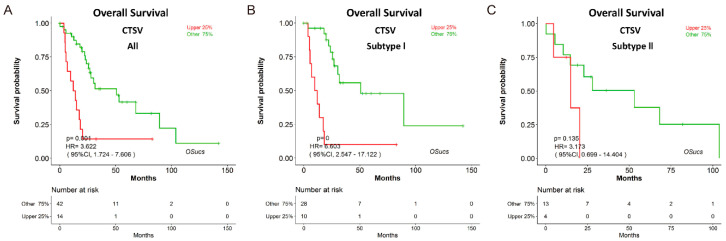
Evaluation of the prognostic value of cathepsin V (CTSV) in OSucs. Kaplan-Meier plots for CTSV in (**A**) All, (**B**) Subtype I, and (**C**) Subtype II UCS, respectively. *p* = 0 denotes *p* < 0.001.

To explore potential prognostic biomarkers for UCS, we evaluated the prognostic abilities of human genes using Cox regression analysis, and identified some predictors which significantly correlated with survival in OSucs by selecting the options under the ‘Molecular subtype’ menu (Figure 4a,c,e). Intriguingly, some genes presented subtype-specific prognostic abilities in OSucs. ETV4 (ETS variant transcription factor 4) is an oncogene and a therapeutic target in various tumors [23,24,25,26]. In OSucs, ETV4 was significantly associated with OS of subtype II UCS patients, but not with subtype I or all UCS (Figure 4b,d,f, Table 3), indicating that ETV4 could be a subtype II-specific prognostic biomarker, which is more aggressive than subtype I with higher malignancy [7]. Next, we evaluated the prognostic abilities of another three genes in OSucs to further identify potential prognostic biomarkers for UCS or its molecular subtypes. Angiopoietin-like protein 4 (ANGPTL4), a newly developed diagnostic and prognostic biomarker, acts as a potential therapy target for renal cell carcinoma, prostate cancer and hepatocellular carcinoma [27,28,29]. Histone cluster 1 H1 family member c (HIST1H1C), an epigenetic regulator, is associated with a poor prognosis in neuroblastoma patients under hypoxia induction [30]. Cathepsin V (CTSV), also known as cathepsin L2, is a lysosomal cysteine peptidase which has an association with poor overall survival of breast cancer [31]. As a result, ANGPTL4, HIST1H1C and CTSV were all significantly associated with OS of all UCS patients (Figure 5, Figure 6 and Figure 7). Interestingly, ANGPTL4 and HIST1H1C were significantly associated with OS of subtype II UCS patients, but not with subtype I (Figure 5 and Figure 6, Table 3). In contrast, CTSV was significantly associated with OS of subtype I UCS patients, but not with subtype II (Figure 7, Table 3). These results indicate that ETV4, ANGPTL4, HIST1H1C and CTSV may be potential prognostic biomarkers for UCS in a molecular subtype-specific manner.

## 4. Discussion

UCS is a type of rare but lethal malignant tumor with high metastasis and recurrence rate [32]. Due to the current limitations in the prognosis of UCS patients [33], it is urgent to develop potential prognostic biomarkers in UCS. One way to do this is to perform analysis on gene expression profiling to identify new biomarkers. In this study, we used a UCS dataset that has RNA-seq and clinical follow-up data from TCGA to establish an online web server, named OSucs. This is the first online prognosis analysis tool to evaluate the association between a candidate gene and survival of UCS patients based on the molecular subtype-specific manner. The limitation of this server is the sample size, as only 56 samples are currently available in OSucs. When more datasets with follow-up information become available, we will update this server to enlarge and improve it for users.

We have evaluated the association between survival and previously published genes for UCS on the OSucs server. Four adverse prognostic markers, including p53 [19], p-flt-1 and VEGFR3 [21,22] and CA19-9 [20], and a beneficial prognostic biomarker, ER [19], have all been confirmed for their risk prediction capabilities in OSucs, indicating the reliability of our web server. As therapeutic targets, HER2 and EPCAM have been reported as carcinogenic factors with high expression in UCS, which correlate with poor prognosis [34]. Further, serum CA125 is a prognostic factor for UCS, the elevation of which predicts the worst survival [5,20]. However, ERBB2 (encoding HER2 protein), EPCAM or MUC16 (encoding CA125 protein) genes have no significant association with survival in OSucs (with *p* value 0.624, 0.922 and 0.928, respectively). This is likely due to the fact that OSucs is based on data from mRNA expression profiling, while these reported prognostic biomarkers are based on protein level detection.

In our previous study, we identified two distinct molecular subtypes of UCS with different gene expression patterns and clinicopathologic characteristics. Remarkably, subtype I UCS recapitalizes low-grade UCS, while subtype II UCS is more likely to be high-grade UCS with higher tumor invasion rate and tumor weight [7]. Therefore, it is necessary to develop new potential prognostic biomarkers to distinguish molecular subtypes of UCS. As a result, the prognostic abilities of these genes are molecular subtype-specific, where ETV4, ANGPTL4 and HIST1H1C are subtype II-specific, while CTSV is subtype I-specific. This indicates that these genes may be potential prognostic biomarkers in a subtype-specific manner, which may be helpful for subtype-specific targeted therapy, especially for higher malignant subtype II UCS. This could ensure that the future targeted treatment of UCS is performed in a subtype-specific manner, as researchers have done for breast cancers in clinics [35,36]. Further risk stratification of molecular subtypes would provide more precise clinical management.

## 5. Conclusions

In summary, we built an online tool to identify prognostic biomarker using expression profiles and clinical data of UCS patients. This platform will facilitate the identification of new prognostic biomarkers and strategies to develop targeted therapies for treating UCS.

## Figures and Tables

**Table 1:** Clinicopathologic characteristics (*N* = 56).

TCGA (The Cancer Genome Atlas)		*N* = 56	Percentage
Overall survival (months)	Range	0.27–142.3	
	Median	20.37	
Disease-free interval (months)	Range	0.27–142.3	
	Median	13.57	
Molecular subtype	I	38	68%
	II	17	30%
	Other	1	2%
Clinical stage	Stage I	21	38%
	Stage II	5	9%
	Stage III	20	36%
	Stage IV	10	18%
History of hormone therapy	Yes	7	13%
	No	28	50%
	Unknown	21	
Therapy outcome	Complete Response	29	52%
	Partial Response	4	7%
	No Response	13	23%
	Unknown	10	
Histological type (NOS: not otherwise specified)	Homologous Type	13	23%
	Heterologous Type	20	36%
	NOS	23	41%
Hypertension	Yes	28	50%
	No	23	41%
	Unknown	5	
Pregnancies	0	4	7%
	1	4	7%
	2	19	34%
	3	15	27%
	4+	7	13%
	Unknown	7	

**Table 2:** Verification of previous published predictors for UCS/uterine cancer (UC) survival in OSucs.

Gene Symbol	Biomarker Name	Clinical Survival Terms	In OSucs					In Reference					Worse Prognosis (Expression)	Ref.
			Cut-Off	*p* Value	HR	95%CI	Case	Cut-Off	*p* Value	Case	Detection Level	Validation		
TP53	p53	OS	Upper 25% (*n* = 14 vs. 42)	0.017	2.542	1.179–5.481	56	Upper *n* = 135/Lower *n* = 38	PFS: *p* = 0.01	173	mRNA		Higher	[19,22]
		DSS		0.014	2.663	1.223–5.797								
		DFI		0.0001	4.571	2.125–9.835								
		PFI		0.001	3.446	1.657–7.165								
ESR1	ER	OS	Lower 25% (*n* = 42 vs. 14)	0.02	2.574	1.16–5.712	56	Upper *n* = 182/Lower *n* = 116	PFS: *p* < 0.001	298	mRNA		Lower	[19]
		DSS		0.015	2.747	1.222–6.177								
		DFI		no significance										
		PFI		no significance										
ST6GALNAC6	CA19-9	OS	Upper 30% vs. Lower 30% (*n* = 17 vs. 17)	0.03	3.107	1.115–8.655	56		DFS: *p* = 0.073	483	serum		Higher	[20]
		DSS		0.017	3.863	1.267–11.776								
		DFI		0.024	3.307	1.17–9.35								
		PFI		no significance										
FLT1	p-flt-1	OS	Upper 30% (*n* = 17 vs. 39)	0.015	2.479	1.191–5.158	56	Upper *n* = 9/Lower *n* = 12	OS: *p* = 0.008	21	protein	Yes, IHC assay	Higher	[21]
		DSS		0.023	2.415	1.129–5.163								
		DFI	Upper 25% (*n* = 14 vs. 42)	no significance										
		PFI		0.049	2.08	1.002–4.316								
FLT4	VEGFR3	OS	Upper 30% (*n* = 17 vs. 39)	0.009	2.657	1.282–5.509	56	Upper *n* = 10/Lower *n* = 29	OS: *p* = 0.052	39	protein	Yes, IHC assay	Higher	[22]
		DSS		0.018	2.483	1.169–5.273								
		DFI		0.044	2.2	1.021–4.743								
		PFI		no significance										

**Table 3:** Evaluation of potential predictors for UCS survival in OSucs.

Gene Symbol	Clinical Survival Terms	In OSucs					Worse Prognosis (Expression)
		Cut-Off	*p* Value	HR	95%CI	Subtype	
ETV4	OS	Upper 25%	0.042	4.324	1.057–17.691	II	Higher
ANGPTL4	OS	Upper 25%	0.007	8.181	1.768–37.86	II	Higher
HIST1H1C	OS	Upper 25%	0.006	11.338	1.973–65.138	II	Higher
CTSV	OS	Upper 25%	<0.001	6.603	2.547–17.122	I	Higher

## Supplementary Materials

The following are available online at https://www.mdpi.com/2073-4425/11/9/1040/s1. Figure S1. Survival analysis (DSS, DFI, PFI) of clinicopathologic characteristic of UCS patients in OSucs. Figure S2. Validation of previous reported prognostic biomarkers (TP53, ESR1 and ST6GALNAC6) in OSucs. Figure S3. Validation of previous reported prognostic biomarkers (FLT1 and FLT4) in OSucs.
