# Supplementary figures and images for: OSucs: An Online Prognostic Biomarker Analysis Tool for Uterine Carcinosarcoma
Source: Genes (Basel). 2020 Sep 3;11(9):1040. doi: 10.3390/genes11091040 (PMC7563768; doi:10.3390/genes11091040)

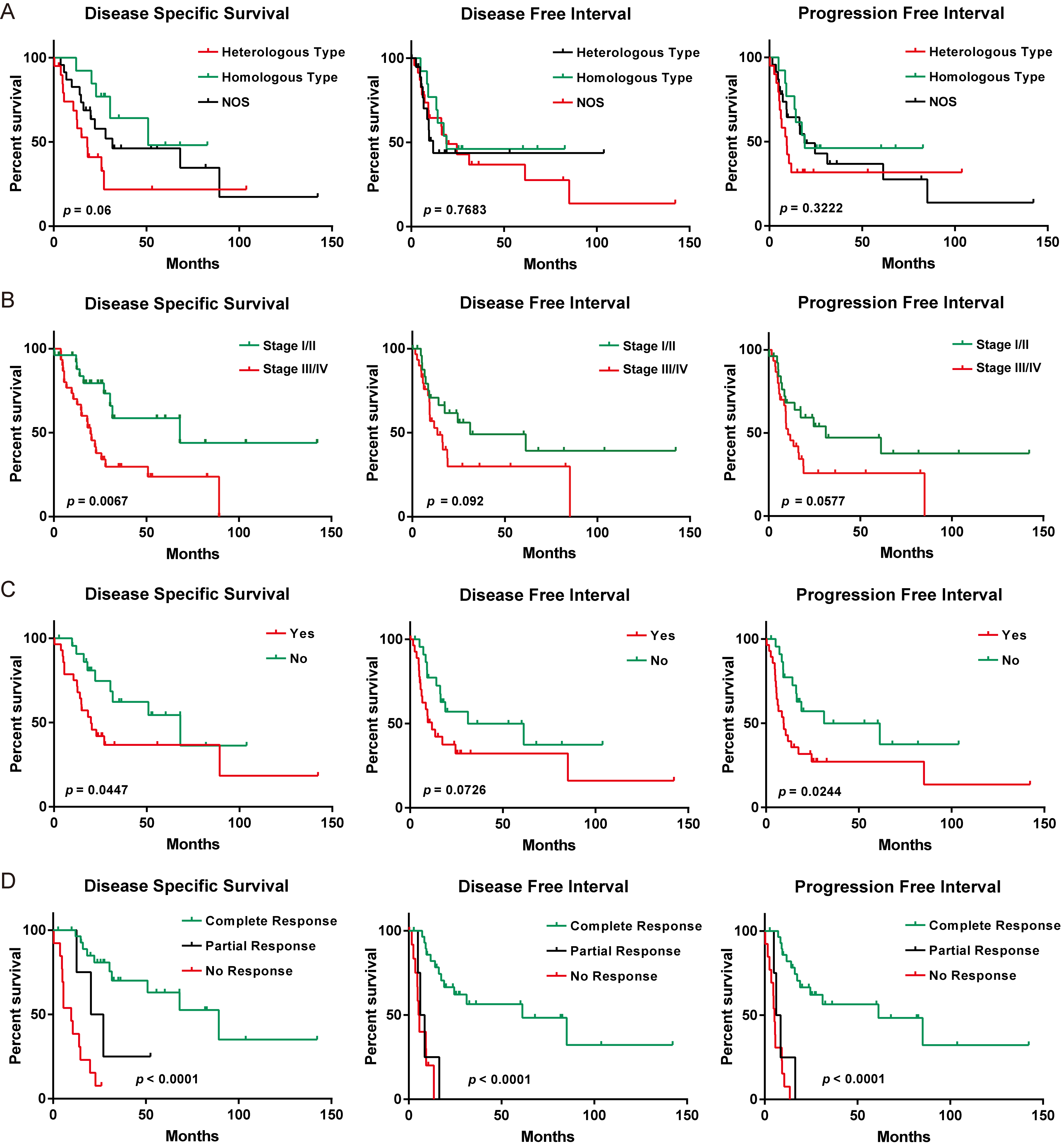

Supplement: Supplementary file 1 [file genes-11-01040-s001.zip › Supplementary Figures/Figure S1.tif]

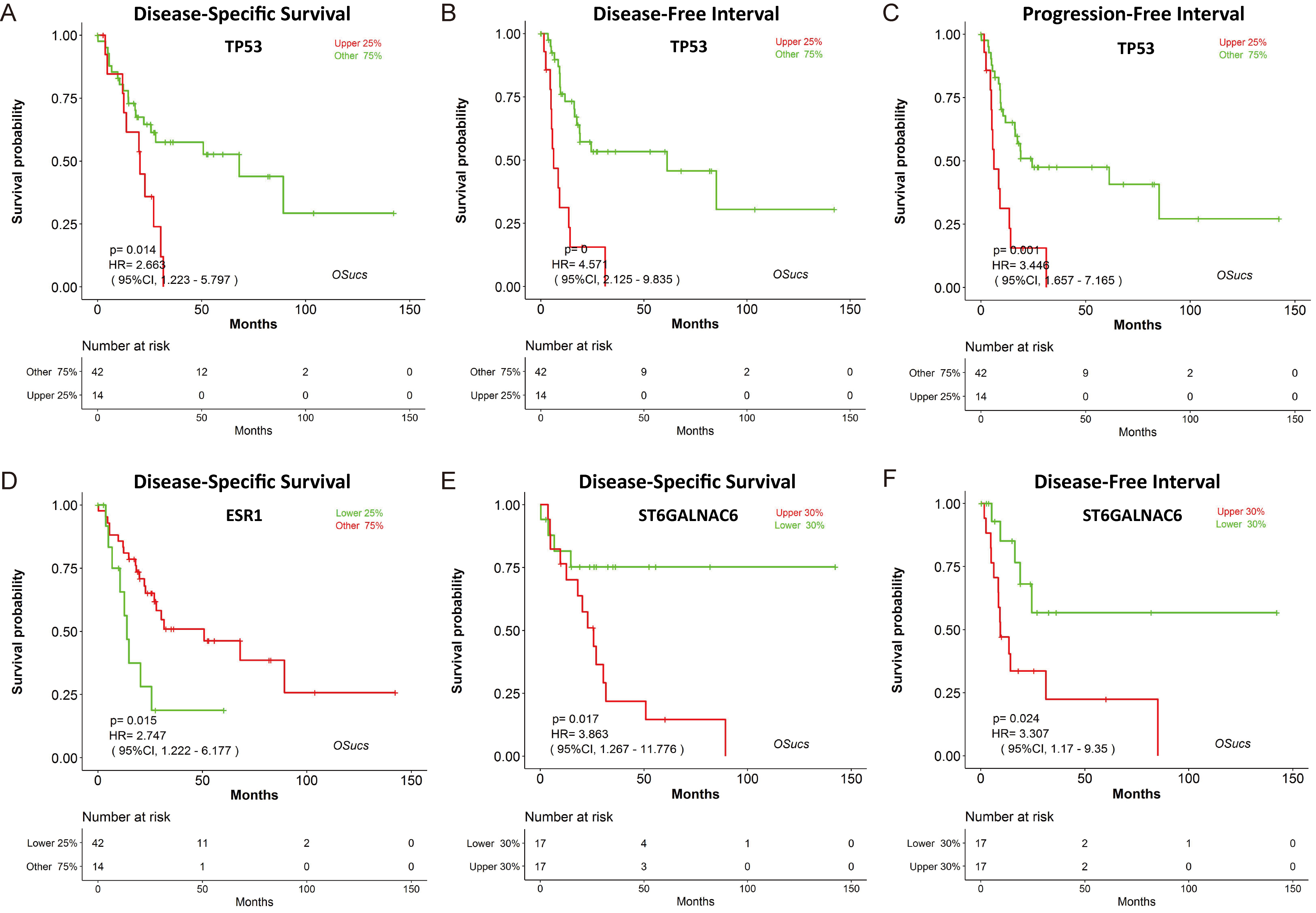

Supplement: Supplementary file 1 [file genes-11-01040-s001.zip › Supplementary Figures/Figure S2.tif]

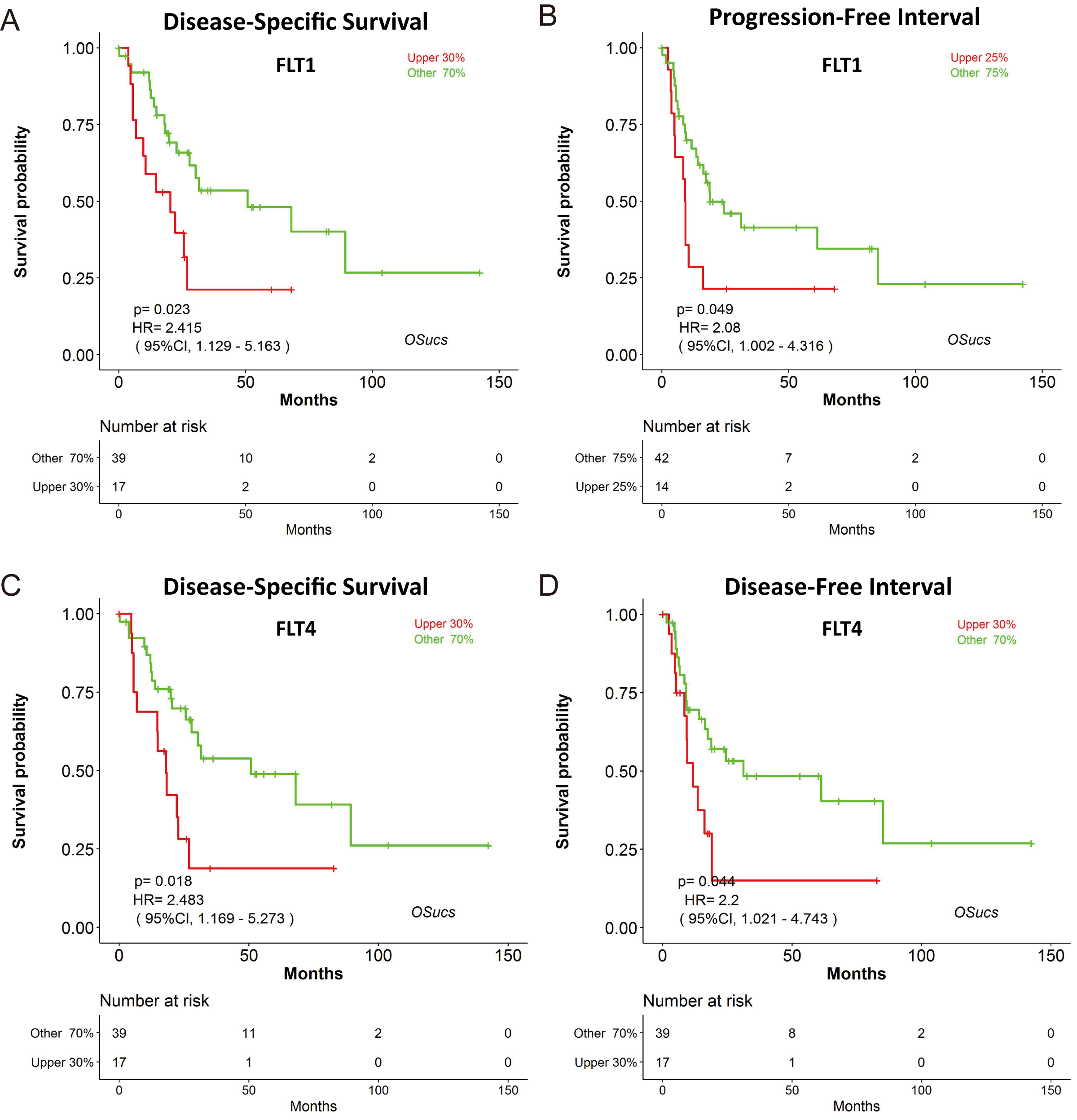

Supplement: Supplementary file 1 [file genes-11-01040-s001.zip › Supplementary Figures/Figure S3.tif]
